# Supercritical Impregnation of Olive Leaf Extract in Poly(L-lactic acid-co-caprolactone) Filaments: An Environmentally Friendly Approach to Obtaining Active Biomedical Materials

**DOI:** 10.3390/polym17111464

**Published:** 2025-05-25

**Authors:** Juan Ramón Montes-Lobato, Noelia D. Machado, Cristina Cejudo-Bastante, Casimiro Mantell-Serrano, Lourdes Casas-Cardoso

**Affiliations:** Chemical Engineering and Food Technology Department, Faculty of Science, Wine and Agrifood Research Institute (IVAGRO), University of Cadiz, 11510 Puerto Real, Spain; juanramon.monteslobato@alum.uca.es (J.R.M.-L.); noelia.machado@uca.es (N.D.M.); cristina.cejudo@gm.uca.es (C.C.-B.); casimiro.mantell@uca.es (C.M.-S.)

**Keywords:** olive leaf extract, supercritical fluid, poly(L-lactic-co-caprolactone) (PLCL), active compound delivery systems

## Abstract

The valorization of by-products in the olive sector has increasingly become the focus of business and research in the context of biorefineries. This work evaluates the recovery of bioactive compounds from olive leaves and their subsequent incorporation into poly(L-lactic- acid-co-caprolactone) (PLCL) filaments through supercritical impregnation. Obtaining an olive leaf extract (OLE) using enhanced solvent extraction at a high pressure (ESE with CO_2_/ethanol 1:1 *v*/*v*) resulted in higher yields and concentrations of bioactives with high antioxidant and anti-inflammatory activity. No significant differences were found between the extracts obtained with different water regimes (irrigated and dry land). The supercritical impregnation of PLCL filaments showed that a low depressurization rate is essential to avoid material deformation, while the impregnation pressure and temperature influenced the OLE loading and antioxidant activity of the filaments. In vitro release studies showed the prolonged release of active compounds over 90 days, and the kinetics best fit the Korsmeyer–Peppas model, suggesting a diffusion mechanism. These results validate supercritical impregnation as a promising strategy for the development of OLE-active PLCL filaments with potential for biomedical applications requiring sustained therapeutic release.

## 1. Introduction

The generation of agri-food waste is a global challenge with profound environmental, human health, and social implications. The valorization of agri-food waste is an essential strategy to move towards a sustainable bio-economy model. From an economic perspective, the production of high value-added products through the integrated use of agri-food by-products is highly beneficial due to the availability and low cost of these by-products. Moreover, the valorization approach is considered compatible with the UN Sustainable Development Goals (SDGs). Many researchers are therefore exploring the recovery and transformation of these wastes into a wide range of bioactive products and compounds, such as food and biomedical additives, biopolymers, bio-based chemicals, bioenergy, and biofuels, with the aim of promoting a sustainable bioeconomy model [1].

The olive (*Olea europaea* L.) industry generates large quantities of solid waste from olive trees, including seeds, pulp, and pruning residues. Significant quantities of olive leaves are generated throughout the year by olive production. On average, each olive tree produces around 25 kg of by-products each year, primarily leaves and branches [2]. Additionally, a significant quantity of leaves is separated from the fruit during the oil extraction process, equivalent to around 10% of its weight [3]. Olive leaves are mainly used for animal consumption, biomass production, or combustion [4,5,6]. However, they are rich in numerous bioactive compounds, such as oleuropein, hydroxytyrosol, oleoside, luteolin, and phenolic acids (i.e., verbascoside), which have demonstrated important biomedical properties, such as antiviral, antimicrobial, anti-inflammatory, antioxidant, and anti-Alzheimer activities [7]. In addition, olive leaves have traditionally been used for medicinal purposes in Mediterranean and European countries, such as Spain, Tunisia, Greece, Morocco, Italy, Turkey, Palestine, and France. They have been particularly useful in treating fever and various infections, including malaria [8]. In some regions, it is consumed in the form of olive tea, prepared with fresh or dried leaves. Therefore, given the valuable profile of olive leaves as a source of compounds with therapeutic properties, sustainable practices to promote the reuse of olive leaf waste are needed.

The first step in recovering bioactive compounds from agri-food by-products is the choice of extraction method. In this respect, new extraction technologies are being developed to contribute to their sustainable valorization. On the one hand, the aim is to increase yields, optimize extraction process parameters, and minimize influences that affect the extraction and degradation of target compounds, as well as to reduce costs and process times. On the other hand, the challenge remains to achieve the best results using methods that do not harm the environment or consumer health, reduce energy costs, and use safe solvents, including CO_2_ and ethanol [9]. High-pressure extraction techniques include pure scCO_2_ extraction, which is mainly used for the extraction of low-polar compounds, and enhanced solvent extraction (ESE), a promising technique that combines the use of pressurized fluids with low-viscosity fluids, such as CO_2_. ESE has been widely used for the extraction of polar compounds, such as flavonoids and polyphenols [10,11]. The use of carbon dioxide reduces liquid solvent consumption and evaporation steps and provides an inert environment during the extraction process, helping to prevent the degradation of sensitive compounds.

Antioxidants are known to have great potential in the effective treatment of diseases such as inflammatory, digestive, neurodegenerative, and cancer diseases. Their beneficial health effects depend largely on their bioaccessibility/bioavailability. However, ensuring their efficacy by maintaining antioxidant concentrations within a therapeutic window is not always possible with conventional dosage forms. Unfortunately, factors such as low aqueous solubility, poor physicochemical stability, and enzymatic instability reduce the bioavailability of most antioxidants [12]. To overcome the limitations of this field, several innovative strategies have been developed in recent decades. These include designing membranes and foams with biological and mechanical properties suitable for tissue replacement, using materials with antimicrobial or antifungal properties to treat infections, developing controlled drug release systems, and using functionalized polymers to promote wound healing [13,14]. Of great interest is the development of controlled drug delivery products, of which the advantage lies in the prolongation of the therapeutic effect through continuous release at the cellular target or at an easily accessible location, while maintaining the concentration between therapeutic and toxicity limits [15].

Various techniques are employed in the production of materials for biomedical purposes. However, some conventional methodologies are limited in their effectiveness or applicability due to shortcomings. For example, polymer-processing methods for tissue engineering applications sometimes require large amounts of toxic solvents, long processing times, or high temperatures, all of which can damage or increase the toxicity of the final product [16,17]. To overcome the limitations of conventional methods, innovative approaches have been developed. One such approach is the impregnation of antioxidant compounds into polymeric matrices using supercritical fluids, which has been shown to offer multiple advantages. These advantages stem from the unique physicochemical properties of supercritical fluids, which are intermediate between those of a gas and a liquid, and their virtually zero surface tension. The most significant benefits of this technique are the high efficiency with which the active ingredient is delivered, its rapid diffusion into the carrier matrix, the ability to control drug loading, and the elimination of organic solvent residues. Supercritical carbon dioxide (scCO_2_), with a critical temperature of 31.1 °C and a critical pressure of 73.8 bar, is a particularly notable fluid in this group due to its non-toxic and non-flammable properties, making it suitable for obtaining formulations and materials free of toxic contaminants [18,19].

Previous results evaluated the impregnation of olive leaf extract (OLE) into biomedical filaments of thermoplastic polyurethane (TPU) and polylactic acid (PLA) in the range of 100–400 bar and temperatures of 35–55 °C. TPU showed an OLE load and antioxidant activity approximately 4 times higher than PLA under optimal operating conditions [20]. Poly(ethylene glycol-co-cyclohexane-1,4-dimethanol terephthalate) (PETG) is a biocompatible, amorphous, and recyclable copolymer that has been successfully used in supercritical fluid impregnation processes. Incorporating OLE using this technique noticeably affects the mechanical behavior of PETG filaments. However, the operating conditions of the supercritical process significantly affect both the tensile strength and elastic modulus [21]. In addition, promising results have been documented for OLE-impregnated poly(lactic-co-glycolic) (PLGA) scaffolds with chitosan via scCO_2_, with foaming and loading levels suitable for use in bone, cardiovascular, or neural tissue regeneration [22]. Advances have also been observed in the design of OLE-impregnated oral dissolution films, resulting in release and dissolution patterns consistent with their high potential for use as antioxidant drugs [23].

Poly(L-lactic-co-caprolactone) (PLCL) is a biodegradable copolymer composed of lactic acid and caprolactone monomers. The glass transition (Tg) and melting temperatures (Tm) of PLCL are variable as they depend directly on the ratio of lactic acid to caprolactone in its composition [24]. The semi-crystalline nature of the polymer, together with its non-toxic profile and good biocompatibility, makes it a material of great interest for various medical applications [25]. The degradation time in the human body is typically between one and three years, although this can vary depending on the proportion of monomers and specific environmental conditions. The ability to control the rate of degradation of PLCL is a critical factor in its medical applications. This allows the design of implants and drug delivery systems that maintain their integrity for a desired period before being naturally eliminated by the body [26]. In the field of tissue engineering, PLCL has been actively investigated for use in soft tissue engineering, including blood vessels, tendons, ligaments, and smooth muscle [27,28,29]. In terms of drug delivery systems, PLCL has been used to deliver dexamethasone, a widely used drug in the treatment of inflammatory and autoimmune diseases, but of which the long-term systemic use is associated with serious adverse effects. The study concluded with an in vivo evaluation using blood and tissue samples from rats after subcutaneous implantation of the implants, which showed that they were safe [30].

Taking into account the potential of PLCL in the biomedical field, as well as the properties of the olive leaf extract, the main objective of the present work is to evaluate the supercritical impregnation of OLE in PLCL filaments. In a first step, the extraction of olive leaves grown under different water regimes (irrigated and dry land) was studied. The best extract was then used to impregnate the PLCL filaments, evaluating the influence of different operating conditions (pressure, temperature, and depressurization rate) on the degree of expansion, loading, and antioxidant activity of the resulting filaments. The best impregnation conditions were selected, the release of the filaments was evaluated over 90 days, and the results were adjusted to the zero order, Higuchi and Korsmeyer–Peppas models. It is expected that the results of this study will promote the use of by-products from the olive sector for producing high-value materials.

## 2. Materials and Methods

### 2.1. Raw Materials and Chemical Reagents

The olive leaves (*Olea europaea* L., “Hojiblanca” variety) from different water regimes (irrigated and dry land) were donated by the Olivarera San José de Lora de Estepa, in the region of Estepa (Seville). The leaves were manually separated from the branches and fruits, dried under ambient conditions, ground to a particle size of ~3 mm, and stored in a moisture-controlled environment away from the light.

The PLCL polymer filaments (1.75 mm in diameter) were supplied by Lattice Services (Loos, France). The molar fractions of the comonomers lactic acid and caprolactone are 70% and 30%, respectively. The minimum Tm and Tg are 150 °C and 35 °C, respectively. Degradation in organisms occurs 12–24 months after implantation.

For extraction and impregnation operations, carbon dioxide (99.99%) was supplied in cylinders by Carburos Metálicos, S.A. (Sant Esteve Sesrovires, Spain), and ethanol (96.49%) was supplied by Alcoholes del Sur, S. A. (Córdoba, Spain). The chemical reagents 2,2-diphenyl-1-picrylhydrazyl (DPPH), lipoxidase from *Glycine max* (≥ 50,000 U/mg), linoleic acid (≥99%), xylenol orange (disodium salt), Na_2_HPO_4_ (≥99%), KH_2_PO_4_ (≥99%), and H_2_SO_4_ (5 N) were supplied by Sigma-Aldrich (Merck KGaA, Darmstadt, Germany). The chemical reagents FeSO_4–_7H_2_O (≥99%), tris(hydroxymethyl)aminomethane (99.8–100.1%), HCl (36.5–38.0%), and methanol (99.9%) were supplied by Panreac Química S.L.U. (PanReac AppliChem ITW Reagents–Reixac, Barcelona, Spain), while the reagents KCl (≥99%) and NaCl (≥99%) were supplied by Panreac Química S. A. (Montcada i Reixac, Barcelona Spain). The analytical-grade solvent, dimethyl sulfoxide (DMSO) for the anti-inflammatory tests, was obtained from Panreac AppliChem (Darmstadt, Germany).

The solvents formic acid (98%), acetonitrile, and dichloromethane (99.9%, stabilized with ~20 ppm of amylene) for UV, IR, HPLC, and ACS quality, were supplied by Panreac Química S.L.U. (PanReac AppliChem ITW Reagents–Reixac, Barcelona, Spain). The analytical chemical standards, oleuropein (≥98.0%), hydroxytyrosol (≥98.0%), and luteolin-7-glucoside (≥98.0%), were provided by Sigma Aldrich (Merck KGaA, Darmstadt, Germany).

### 2.2. High-Pressure Extraction

**Figure 1:**
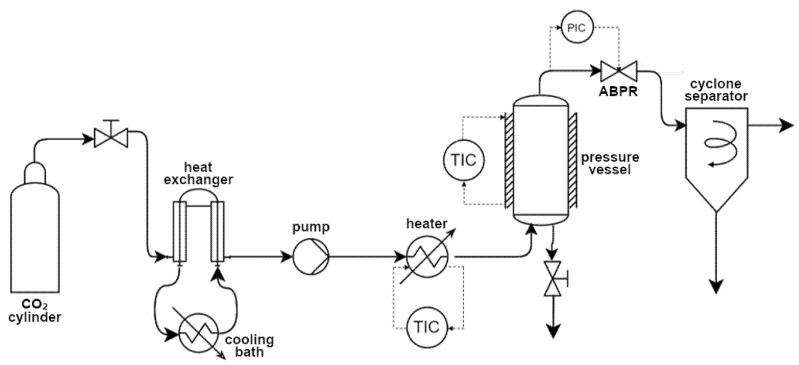
Flow diagram of the extraction/impregnation system used (ABPR: automatic back pressure regulator, T: temperature, P: pressure, TIC: temperature integrated controller, PIC: pressure integrated controller).

The extractions were carried out in a supercritical extraction plant fitted with a 1000 mL vessel (model SF1000 Thar Technology, Pittsburgh, PA, USA). A simplified diagram of the equipment can be seen in Figure 1.

For the extraction tests, a filter paper cartridge was filled with ~50 g of dried olive leaves (irrigated and dry land) and placed in the pressure vessel. First, an extraction with pure scCO_2_ (120 bar and 80 °C) at a mass flow of 10 g/min was performed for 3 h to remove low polar compounds. This was followed by a second extraction at 120 bar and 80 °C using a CO_2_:ethanol mixture (1:1 *v*/*v*) in batch mode for 24 h (ESE) [31]. Previous work has shown that in order to achieve an adequate richness of the extract in the bioactive compounds of interest, which are generally polar, it is advisable to first remove a large part of the non-polar compounds [32]. Each extraction condition was carried out in triplicate (*n* = 3) for robustness and reliability in the experimental results.

The global extraction yield was determined according to Equation (1).

(1) $$\%\;Extraction\;yield=\frac{m_{e}\;(g)}{m_{o}\;(g)}\times 100$$

where *m_e_* is the mass of the dried extract and *m_o_* is the initial mass of olive leaves.

### 2.3. Characterization of the Extracts

#### 2.3.1. Antioxidant Activity

The antioxidant activity of the extracts was assessed using the DPPH assay, in accordance with the protocols established by Brand-Williams et al. [33] and Scherer and Godoy [34]. This method quantifies antioxidant activity by measuring the color change of the samples, which is caused by the reduction of the DPPH free radical and is indicated by a decrease in absorbance at 515 nm. This spectrophotometric variation is a consequence of the interaction between the bioactive compounds present in the extracts and the DPPH radical. Decreases in absorbance were measured using a Synergy™ HTX Multi-Mode Microplate Reader Spectrophotometer (Agilent Technologies, Inc., Santa Clara, CA, USA).

Each OLE sample was diluted at 60 to 7000 µg/mL in ethanol; 7 µL from each dilution sample was added to 293 µL of 6·10^−5^ M DPPH solution. After 2 h of incubation at room temperature in the dark, the absorbance was measured against a blank of ethanol. By comparing the initial DPPH absorbance (*Abs*_0_) against the 2 h absorbance measured (*Abs_f_*), the percentage of inhibition (*% I*) was determined according to Equation (2). All experiments were performed at room temperature in the absence of light in triplicate.

(2) $$DPPH\;\%\;I=\frac{{Abs}_{0}-{Abs}_{f}}{{Abs}_{0}}\times 100$$

The effective concentration (IC₅₀), defined as the concentration of extract required to reduce the absorbance of the DPPH free radical by 50%, was determined via graphical analysis. This involved plotting the DPPH% I against the extract concentration. The results obtained were expressed as the Antioxidant Activity Index (AAI) according to Equation (3):

(3) $$AAI=\frac{C_{DPPHf}}{{IC}_{50}}$$

where *C_DPPHf_* is the final concentration of DPPH. The assays were carried out in triplicate.

#### 2.3.2. Anti-Inflammatory Capacity

The anti-inflammatory capacity was measured in duplicate by adapting the method described by Pinto et al. for determining lipoxygenase activity using the ferric ion–xylenol orange (FOX) complex oxidation [35].

First, the reagents were prepared: original tris-HCl buffer (50 mM at pH 7.4), linoleic acid buffer (140 µM in original tris-HCl buffer), FOX reagent (30 mM H_2_SO_4_, 100 µM xylenol orange, 100 µM FeSO_4_ in 90% *v*/*v* methanol/H_2_O solvent), and lipoxygenase buffer (10,000–20,000 U/mL in original tris-HCl buffer). OLE sample solutions (from 50 to 1140 μg/mL) were dried to remove ethanol and diluted in a 10% *v*/*v* DMSO aqueous solution.

For each sample, one test well and one blank well were prepared in triplicate. In all control wells, 20 µL of the original buffer, 20 µL of a 10% *v*/*v* DMSO aqueous solution, and 40 µL of the buffered lipoxygenase solution were added. In the sample wells, 20 μL of the aqueous solvent was replaced by the corresponding OLE sample solution.

Then, 40 µL of the buffered linoleic acid solution was added and left to react for 25 min. After that, 100 µL of FOX reagent was added, and after 30 min, the absorbance in the test wells was measured at 560 nm (*Abs_t*0*_* for the control and* Abs_t_* for the sample).

All experiments were performed at room temperature in the absence of light in triplicate.

Results are expressed as the percentage of inhibition (*% I*) according to Equation (4),

(4) $$\%\;I=1-\frac{\left({Abs}_{t}-{Abs}_{b}\right)}{\left({Abs}_{0}-{Abs}_{0b}\right)}\times 100$$

where *Abs_t_* is the absorbance of the test sample, *Abs_b_* is the absorbance of the *blank* of the test sample, *Abs*_0_ is the absorbance of the control, and *Abs*_0*b*_ is the absorbance of the blank of the control.

The IC_50_, defined as the concentration of extract required to reduce linoleic acid by 50%, was calculated graphically by plotting the *% I* versus the concentration of the extract.

#### 2.3.3. Identification and Quantification of Major Phenolic Compounds via High-Performance Liquid Chromatography Coupled with a Diode Array Detector (HPLC-DAD)

The quantification of the main phenolic compounds present in OLE was performed using HPLC with a system supplied by Jasco Corporation (Madrid, Spain). Chromatographic separation was performed using a Fortis C18 column (150 mm × 3 mm internal diameter, 5 μm particle size), which was supplied by Fortis Technologies Ltd. and distributed in Spain by Jasco Corporation, Madrid, Spain.

Chromatographic analysis was performed using a binary gradient system consisting of a 0.1% *v*/*v* aqueous solution of formic acid (mobile phase A) and a 0.1% *v*/*v* solution of formic acid in acetonitrile (mobile phase B). The analysis was conducted at a constant flow rate of 0.6 mL/min and a temperature of 20 °C. The elution profile started with 0% mobile phase B, increasing to 5% after 0.05 min, and maintained until 10 min. Then, it increased to 20% B at 20 min and 30% B at 25 min, and this composition was maintained for 3 min. After that, it increased to 50% B at 46 min and reached 100% B at 52 min. The gradient then decreased to 50% B at 57 min, finally reaching 5% B at 60 min [36]. Samples were automatically injected after a delay of 5 min using a fixed volume of 15 μL. Data acquisition and processing were performed using JASCO ChromNAV software (version 2.04.04, JASCO).

Oleuropein and hydroxytyrosol were identified at 280 nm with retention times of 22.58 and 5.81 min, respectively. Luteolin-7-glucoside was detected at 340 nm with a retention time of 20.96 min. The compounds were quantified spectrophotometrically using calibration curves prepared at concentrations between 10 and 200 mg/L diluted in solvent A. These curves are represented by Equation (5) (oleuropein), Equation (6) (luteolin-7-glucoside), and Equation (7) (hydroxytyrosol),

(5) $$A_{ol}=7590.7\times C_{ol},\;R^{2}=0.9978$$

(6) $$A_{lu}=57975\times C_{lu},\;R^{2}=0.9984$$

(7) $$A_{hi}=7200.5\times C_{hi},\;R^{2}=0.9988$$

where A_ol_, A_lu_, and A_hi_ are the areas under the peak, and C_ol_, C_lu_, and C_hi_ are the concentrations (µg/mL) for oleuropein, luteolin-7-glucoside, and hydroxytyrosol, respectively.

### 2.4. Supercritical Solvent Impregnation

The impregnation experiments were performed using a system supplied by Waters Corporation (Milford, CT, USA). This system was equipped with a 100 mL thermostatted stainless steel cell, a P50 double-piston CO_2_ pump, and an automatic back-pressure regulator (ABPR). The equipment’s design is analogous to that depicted in Figure 1.

For each test, 3 mL of OLE was introduced into the bottom of the cell. Four 25 mm-long PLCL filament fragments were then placed on top of the extract using a metal support. This was intended to prevent direct contact between the filaments and the liquid extract. A CO_2_ flow rate of 10 g min^−1^ was then applied until the desired pressure was reached. Impregnation was carried out in batches lasting 1 h, with each experimental condition replicated in duplicate.

Once the process was finished, the filaments were carefully wiped with a wet wipe to remove any extract residue from the surface. The impregnated samples were then stored in the dark to preserve their integrity until use.

Multi-factorial experimental designs with 2 factors, pressure and temperature, with 3 levels for each factor, were performed according to Table 1. The impregnation time (1 h) and the depressurization rate were kept constant in accordance with the corresponding design of the experiment.

The pressure and temperature values were selected to cover a wide range of scCO_2_ density values. At 35 °C, the CO_2_ density increases to 712.81, 901.23, and 972.26 kg/m^3^ at 100, 250, and 400 bar, respectively, at 55 °C, from 325.07 and 810.65 to 906.77 kg/m^3^ at 100, 250, and 400 bar, respectively, and, at 75 °C, from 233.43 and 711.61 to 839.93 kg/m^3^ at 100, 250, and 400 bar, respectively [37]. The range of depressurization rates was determined in order to avoid the physical damage in the impregnated filaments.

### 2.5. Characterization of the Impregnated Filaments

#### 2.5.1. Swelling of the Impregnated Filaments

This study evaluated structural changes in polymer filaments after exposure to scCO_2_, quantifying the degree of permanent swelling or expansion of the polymer at the end of the treatment. To this end, the filament’s volume was measured before impregnation (*V*₀) and several days after its completion (*Vf*), allowing for the diffusion of residual CO_2_ and the stabilization of permanent swelling [37]. The expansion percentage was calculated using Equation (8).

(8) $$\%S=\frac{V_{f}-V_{0}}{V_{0}}\times 100$$

The filaments’ expansion was quantified in triplicate.

#### 2.5.2. OLE Loading of the Impregnated Filaments

The total amount of OLE impregnated in the PLCL samples was determined via gravimetric analysis (Equation (9)). The mass of each sample was recorded before the impregnation process (*m*₀) and after its completion (*mf*). The final masses were re-measured several days after treatment, in order to allow for the complete release of residual CO_2_ trapped in the polymer matrix.

(9) $$\%\;L=\frac{m_{f}-m_{0}}{m_{0}}\times 100$$

#### 2.5.3. Antioxidant Capacity of Impregnated Filaments

A known quantity of impregnated filaments was immersed in 4 mL of a DPPH solution with a concentration of 60 μM, and the decrease in absorbance was monitored at a wavelength of 515 nm over a period of 21 h. *DPPH% I* was calculated according to Equation (2).

#### 2.5.4. Anti-Inflammatory Capacity of Impregnated Filaments

A volume of 20 µL of the 60-day release samples of the polymers impregnated at 75 °C and 100 bar, 250 bar, and 400 bar was subjected to the same procedure as in Section 2.3.2. In each case, the *% I* was calculated according to Equation (4).

#### 2.5.5. Identification and Quantification of OLE Polyphenols in Impregnated Filaments

A piece of impregnated filaments (approximately 50 mg) was dissolved in about 1 mL of dichloromethane in order to dissolve the polymer and release the impregnated compounds. Then, the solvent was completely dried under a vacuum at 45–50 °C using a Heidolph rotary evaporator Laborota 4000 provided by Heidolph Scientific Products GmbH (Schwabach, Germany). After drying, the samples were redissolved in 1 mL of ethanol (polyphenols solvent) and filtered using a nylon syringe filter with a 0.22 μm pore size. Finally, the polyphenols were quantified using the HPLC-DAD method described in Section 2.3.3.

The concentration of polyphenol impregnated was expressed as μg/100 mg of polymer.

#### 2.5.6. Scanning Electron Microscopy (SEM) of Impregnated Filaments

To examine the morphology of the polymer before impregnation and detect any visible OLE particles on the surface of the impregnated filaments, a visual analysis was carried out using SEM. To this end, the samples were fixed to a metal support and coated with a conductive gold layer approximately 15 nm thick. An analysis was performed using a Quanta 200 scanning electron microscope (FEI, Hillsboro, OR, USA) operating at an accelerating voltage of 20 kV in a vacuum.

### 2.6. In Vitro Release Studies

To analyze the release kinetics of the active compounds, a fragment of an impregnated filament (approximately 50 mg) was immersed in 1.5 mL of phosphate-buffered saline (PBS). The PBS solution was prepared by dissolving 1.43 g/L of Na_2_HPO_4_, 0.24 g/L of KH_2_PO_4_, 8 g/L of NaCl, and 0.2 g/L of KCl and then adjusting the pH to 7.4 with NaOH or HCl [38]. The samples were kept at rest in a hermetically sealed container at 37 °C for 1, 2, 5, 10, 30, 60, or 90 days. A separate sample was prepared for each time point.

The study focused on samples impregnated under three selected experimental conditions: 100 bar/75 °C, 250 bar/75 °C, and 400 bar/75 °C. The total amount of polyphenols released was quantified via HPLC, according to the procedure described in Section 2.3.3. Prior to injection, the release medium was filtered using a nylon syringe filter with a pore size of 0.22 µm.

The release rate of the compounds was expressed as the cumulative mass released over time (*m_t_*) relative to the total mass of impregnated compounds (*m*_0_), which corresponds to the value achieved under infinite release conditions according to Equation (10); *m_∞_* was determined as described in Section 2.5.5.

(10) $$Drug\;released=Q_{t}=\frac{m_{t}}{m_{0}}=\frac{m_{t}}{m_{\infty }}$$

### Application of Compound Release Data to Mathematical Models

Zero-order kinetics are characterized by an active compound release rate independent of its concentration, as represented in Equation (11).

*C_t_* = *C*_0_ + *K*_0_
*t* (11)

where *C_t_* is the concentration of active compound released at a given time (t), *C*_0_ is the initial concentration of the active compound, and *K*_0_ is the zero-order rate constant.

Higuchi’s model results from accepting that the release is controlled by the diffusion of the compound through the polymer matrix, assuming, therefore (i) that its concentration in the polymer matrix is several orders of magnitude higher than its solubility, (ii) that no erosion or degradation of the polymer occurs, and (iii) that the release medium acts as a perfect sink [39]. Thus, a pseudo-steady state would be reached for which the concentration would correspond to the saturation concentration of the compound in the medium. Equation (12) is derived from the resolution of Fick’s law and describes the process [39].

(12) $$Q_{t}=k_{H}\sqrt{t}$$

Here, *Q_t_* represents the proportion of compound released (ratio of mass released to mass impregnated), and *k_H_* is the Higuchi constant, which groups together the effects of diffusivity, capillarity, porosity, and solubility [40].

The Korsmeyer–Peppas (also known as potential law) model represents an empirical generalization of the Higuchi model, whereby the transport exponent is calculated by fitting to experimental data and provides some information on the release mechanism.

For a cylindrical geometry, the drug release mechanism follows a Fickian diffusion when *n* < 0.45. The diffusion occurs via case II transport, leading to the zero-order release when *n* = 1; and when the *n* value is between 0.45 and 1, non-Fickian diffusion occurs [41]. The model equation is represented in Equation (13).

(13) $$Q_{t}=k_{KP}\;t^{n}$$

where *k_KP_* is the model constant that incorporates structural and geometric features [40], and *n* is the transport exponent.

### 2.7. Statistical Analysis

All experiments and analytical determinations were repeated in triplicate (*n* = 3), and the results are shown as the mean ± standard deviation.

An analysis of variance (ANOVA) was used to compare the regression curves obtained from dry and irrigated samples, both in the antioxidant activity evaluation method and in the anti-inflammatory activity method. This approach assessed whether there were significant differences in the slopes and/or intercepts of the fitted lines for each type of sample, allowing for the identification of whether the water regime (dry or irrigated) significantly influenced the relationship between the variables studied.

In multifactorial experimental designs, the effect of each factor on the response variables is statistically evaluated using an analysis of variance (ANOVA). To visualize the magnitude and relative importance of these effects, a Pareto diagram was used to represent the different factors, indicating, with the corresponding sign, whether the effect attributed to each experimental variable was positive or negative.

For all the statistical analyses, the results were considered significant at the 95% confidence level (α = 0.05).

The experimental data were processed using the software application Statgraphics Centurion XIX (StatPoint Technologies, Inc., Princeton, NJ, USA).

## 3. Results and Discussion

### 3.1. Enhance Solvent Extraction

Fractionation was carried out by first performing an extraction of olive leaves with pure scCO_2_ followed by another extraction with high proportions of ethanol (CO_2_/ethanol 1:1 *v*/*v*) under subcritical conditions (ESE), according to Section 2.2. This fractionation aims to remove low-polar compounds with low antioxidant and anti-inflammatory potential, such as waxes, fatty acids, or certain carotenoids [42] and to achieve a second fraction of higher purity in the bioactive molecules of interest (flavonoids and polyphenols). Table 2 shows the results of the extraction yields, the chemical and biological quantification of both fractionation stages for each OLE sample (from “dry” and “irrigated” lands).

When the process was carried out with pure scCO_2_, low yields were obtained, around 1%, whereas when ethanol was added to the system, yields reached values above 22% for ESE. Previously reported yields with pure scCO_2_ indicate similar or slightly higher values compared to those obtained in this study. The OLE yield reported at 300 bar and 50 °C was 1.0% [43], 2.8–3.5% at 250 bar and 80 °C and depended on the drying temperature of the leaves [44], and 0.7–2.1% at 100–300 bar and 50 or 100 °C [45]; at 450 bar and 40 °C, it was up to 0.6% [46], and at 300 bar and 35–90 °C, the authors found the highest yield of 4.3% at 90 °C, which decreased significantly to 2.1% at 35 °C [47]. The observed differences can be attributed to the parameters of the extraction process (pressure, temperature, operating time, and solvent flow rate), the moisture content, and the pre-treatment applied [48]. Additionally, agronomic factors such as the olive variety, growing conditions, and harvesting time significantly influence the outcome.

Machado et al. [20] reported an extraction yield with ESE of 13 ± 1% at the same pressure and temperature conditions as those used in this work, despite using extraction times of only 3 h. Later studies reported lower yields (10.7 ± 0.9%) when the extraction time was increased to 24 h, for the same pressure and temperature conditions [21]. These same studies report an extract concentration of 0.11 ± 0.01 g/mL [20] and 0.12 g/mL, higher, in both cases, than that obtained experimentally, as shown in Table 2. The difference in yield is likely due to the pre-treatment of the biomass, since in this work, the leaves were separated from the rest of the pruning residues, with a negligible content of the compounds of interest.

Oleuropein is the major secoiridoid present in olive leaves, and it is known as an antibacterial, antioxidative, antiviral, antiatherogenic, cardioprotective, and antihypertensive agent [45,49]. In the present study, oleuropein was not detected in the pure scCO_2_ extracts; however, it is reported that the supercritical extract obtained at 300 bar and 100 °C with pure scCO_2_ contained 0.19 mg oleuropein/100 g OLE [45], and the extract obtained at the same pressure but 90 °C contained 1.57 mg oleuropein/100 g dry OLE [47]. It should be noted that the latter study, as mentioned above, worked in the range of 35–90 °C and did not detect oleuropein, hydroxytyrosol, or tyrosol in OLE samples obtained with pure scCO_2_ at 35, 50, and 70 °C, so the results are in agreement with those reported in this work. In both cases, the values were low and increased considerably when ethanol was added as a co-solvent, reaching concentrations of 715 mg oleuropein/100 g OLE and 187.82 mg oleuropein/100 g OLE, respectively. However, the reported oleuropein concentrations are much lower compared to those obtained in this work when using ESE. According to Table 2, when a high proportion of ethanol is added to CO_2_, oleuropein levels ranged from 1480 mg oleuropein/100 g OLE to 1750 mg oleuropein/100 g OLE, confirming the hypothesis that increasing the polarity of the extraction system favors the extraction of antioxidant compounds, including oleuropein. The same trend was observed for luteolin-7-glucoside and hydroxytyrosol.

Cejudo Bastante et al. [50] reported oleuropein concentration values of 1275.8 ± 79.5 µg/mL, similar to the results obtained in the present work; however, they informed the concentration of luteolin-7-glucoside at 141.2 ± 11.8 µg/mL and 55.10 ± 1.4 µg/mL for hydroxytyrosol, values considerably higher than those obtained in the current work. This difference may be due to modifications in the extraction method, since in that study, a mixture of CO_2_/ethanol 1:1 *v*/*v* was pumped continuously, unlike the discontinuous mode of operation used in this work.

Plant extracts are a natural alternative to synthetic antioxidants because they have comparable or even superior antioxidant activity [51]. In this context, the antioxidant activity of OLE was evaluated using the DPPH method. According to the classification of Scherer and Godoy [34], the OLE extracts presented high antioxidant capacities since the AAI values were 1 < AAI < 2. Moreover, no significant differences were observed between the results obtained from irrigated and dry systems. The AAI values obtained experimentally were similar to those reported by other authors, such as 1.048 ± 0.1 [52] or 1.06 ± 0.1 [31].

**Figure 2:**
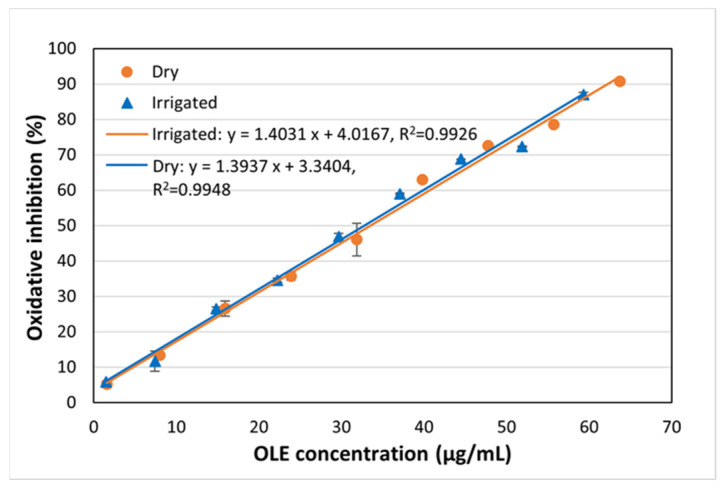
DPPH antioxidant activity of the obtained OLE from lands of different water regimes (dry and irrigated). The results are presented as means and standard deviations (*n* = 3).

Figure 2, on the other hand, shows the % I for the different concentrations tested in both samples, showing that the behavior between irrigated and dry samples is very similar. A statistical analysis was carried out to compare both curves. Table 3 shows the ANOVA analysis obtained, where it is observed that the *p*-value for the cut-off point and for the slope is greater than 0.05 in both cases, so that, at 95% confidence, it is concluded that both regression models are similar. There are therefore no differences in the DPPH antioxidant activity when extractions are carried out using olive leaves from irrigated or dry lands.

**Figure 3:**
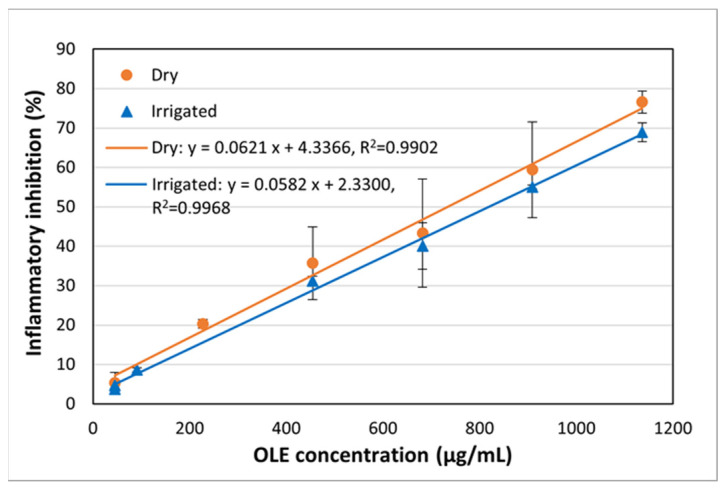
Anti-inflammatory capacity of the obtained OLE from lands of different water regimes (dry and irrigated). The results are presented as means and standard deviations (*n* = 3).

The range of concentrations used in the study of the anti-inflammatory activity of the extracts is wider than that used to determine the antioxidant activity, ranging from 50 to 1140 μg/mL (Figure 3). A similar statistical study (Table 4) allows us to conclude that the behavior of the dry and irrigated samples shows similar anti-inflammatory activity. Again, the *p*-value for the cut-off point and the slope is greater than 0.05, indicating that no statistically significant differences can be affirmed at a confidence level of 95%.

This method of determining anti-inflammatory activity using the FOX lipoxygenase enzyme has been applied to extracts of *Sorbus aucuparia* L. (rowan) obtained with different solvents. The results have shown a range of IC_50_s, depending on the solvent used, between 2 and 9 μg/U [53]. Transforming the data obtained in this work to these units gives a value of 0.108 μg/U, which would imply that the OLE obtained in this work has a higher anti-inflammatory capacity than the extracts evaluated. No data have been found in the literature using this same method to evaluate this activity in OLE.

After characterizing the ESE extracts in terms of the extraction yield, oleuropein, luteolin-7-glucoside, and hydroxytyrosol concentrations, and antioxidant and anti-inflammatory activity, it was found that there were no significant differences between irrigated and dried extracts, so these extracts were used interchangeably in the impregnation of polymers with biomedical applications.

### 3.2. Impregnation into the PLCL Filaments

In the supercritical impregnation process, the nature of the polymer and the extract must be considered, as well as how they interact with supercritical carbon dioxide (scCO_2_). Key parameters in this process include the solubility of the extract’s active compounds in supercritical CO_2_, the polymer’s ability to absorb and expand with supercritical CO_2_, and the affinity between the active compounds and the extract. As these parameters vary depending on the operating conditions used, it is essential to evaluate the effect of changes in pressure and temperature on the loading and biological activity of the new materials generated.

**Figure 4:**
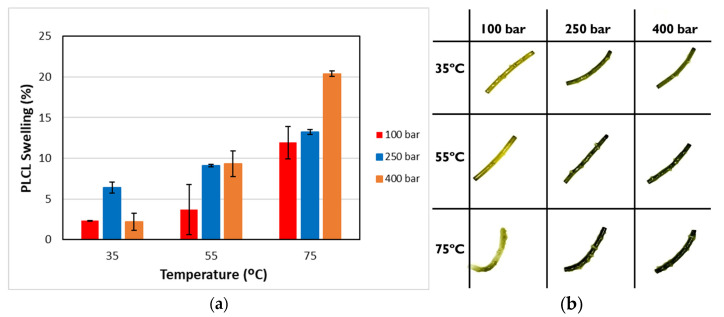
(**a**) Swelling and (**b**) external appearance of the impregnated filaments when operated at a high depressurization rate. The swelling results are presented as means and standard deviations (*n* = 3).

Under supercritical conditions, the absorption of scCO_2_ causes the polymeric chains to reorganize. During the subsequent depressurization stage, the CO_2_ reverts to a gaseous state and is released from the polymer matrix, creating structural voids. This increase in free volume can lead to the polymer swelling permanently [40]. Figure 4a illustrates the degree of permanent swelling observed in PLCL filaments subjected to various temperature and pressure combinations at a high depressurization rate.

The swelling effect increased with pressure, a phenomenon that was particularly noticeable at temperatures of 55 °C and 75 °C. Similarly, under isobaric conditions, increasing the temperature was found to accentuate the swelling of the polymer. Consequently, at the higher analyzed temperatures, significant swelling and foaming occurred, resulting in structural deformation of the polymer material. These differences could be explained by a higher plasticizing effect at 75 °C, given that the polymer’s glass transition temperature is 35 °C according to the manufacturer (Lattice Services, Loos, France). The results described coincide with those reported by [40] for the swelling of polylactic acid (PLA).

To determine the statistical significance of each variable involved, the analysis of the proposed design of the experiment is performed. Table 5 shows the analysis of variance and standardized Pareto plot respectively for the swelling. Temperature is the most influential factor and shows a positive effect on swelling.

Figure 4b shows the physical appearance of the polymers after the impregnation process. An appreciable difference can be observed in the color acquired by the polymers after the impregnation process, as the initial polymer is translucent and colorless. The appearance of irregularities on the surface of the polymer is also observed, most likely as a consequence of bubble growth and rupture upon pressure reduction [54], which affected all the operating conditions studied. The irregularities may be due to the pressure difference between the inside of the polymer and the external medium, probably causing the distribution of the extract along the polymer fragment to be inhomogeneous. This would explain why no reproducible results were obtained in the antioxidant activity assay.

This phenomenon of irregularity formation within the polymer matrix has been analyzed in previous research. Particularly, studies related to the impregnation of contact lenses, where any heterogeneity is also detrimental to the optical properties, so the formation of bubbles, associated with operating at a high depressurization rate, motivates one to change the process using a low rate to avoid these alterations [55]. A suitable depressurization rate seems to be 0.6 bar/min [56], which would translate to 11 h 7 min for 400 bar (highest pressure used).

**Figure 5:**
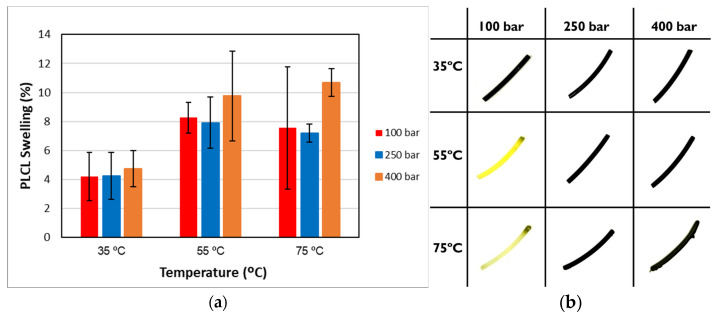
(**a**) Swelling and (**b**) external appearance of the impregnated filaments at low depressurization rate. The swelling results are presented as means and standard deviations (*n* = 3).

Thus, the entire experimental design was repeated using a low depressurization rate, setting a total depressurization time of 22 h to ensure that no breakage of the polymer occurred. The swelling and physical appearance of the impregnated polymers in the second experimental design can be seen in Figure 5. A reduction in swelling to values below 12% is observed (Figure 5a). Table 6 shows the results of the statistical analysis, with the temperature again being the most influential factor on the response variable. It can be seen that, by eliminating the phenomenon that created irregularities, the surface is smooth and the distribution of the extract is apparently homogeneous along the filaments (Figure 5b). However, after depressurization, CO_2_ is still retained inside the polymer, which does not easily escape to the outside, and its diffusion to the outside takes place very slowly.

**Figure 6:**
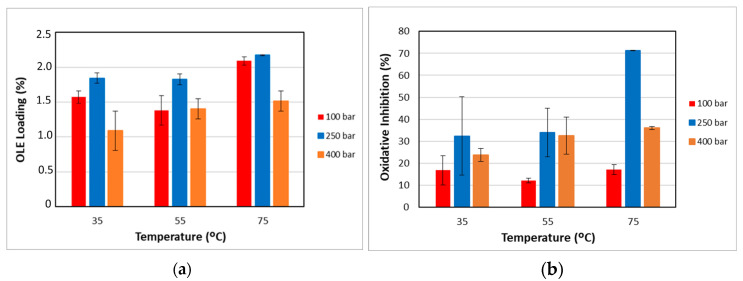
(**a**) OLE loading and (**b**) DPPH antioxidant activity on the impregnated filaments when operated at a low depressurization rate. The results are presented as means and standard deviations (*n* = 3).

Figure 6a shows the loading results of the impregnated polymer under the different pressure and temperature conditions studied. The impregnation percentage (% L) varies between 1.09% and 2.17%, exceeding, for most of the conditions studied, the data reported for the supercritical impregnation of PLA filaments with OLE [20], although they are lower than those of PETG impregnation with OLE, up to 7% [21], or those of TPU impregnation with OLE, up to over 5% [20].

High impregnation loading is obtained when the partition coefficient of the active compound favors the polymer matrix over the supercritical fluid phase [57]. Generally, an increase in the pressure improves both the solvent capacity of scCO_2_ given by an increase in the solubility of the active compounds, as well as the mass transfer phenomenon (viscosity and diffusivity), which improves the diffusion of the active compounds in the polymer matrix [58]. At 250 bar, where the highest loadings are obtained, it seems that the effect of diffusion of the active compounds into the matrix, with their subsequent retention in it, prevails over the increase in the solubilization of the compounds in the scCO_2_ phase. On the other hand, at 400 bar, the prevailing factor is the increase in the affinity of the compounds for the supercritical phase, given by the greater increase in the density of this fluid.

On the other hand, under isobaric conditions and at 75 °C, the density of scCO_2_ is the lowest, which makes the active compounds present a higher affinity with the matrix than with the supercritical phase. This, in turn, results in an increase in the impregnation yields. Higher temperatures also enhance diffusivity, which favors the phenomena of matter transfer into the polymeric phase, with the highest loading values being found in general.

To determine the statistical significance of each variable involved, the analysis of the proposed design of the experiment is performed. Table 7 shows the analysis of variance and standardized Pareto plot, respectively, for the % loading.

These show that the statistically significant effects at a 95% confidence level are the simple effect of temperature (positive), the simple effect of pressure (negative), and the quadratic effect of pressure (negative). This indicates that the extract loading in the polymer increases with temperature and decreases overall with pressure, although with a negative quadratic component that causes intermediate values of pressure to reach the highest loading values. As this is a complex system and difficult to predict due to the multiplicity of factors involved in the interactions among the polymer, solvents, and solute, these trends can explain, to some extent, what the predominant behaviors are at the global level.

This behavior has also been described, among others, for mango leaf extract in PLA [59], where a similar quadratic effect of pressure was found to be significant. For its part, the effect of temperature has been documented in investigations, such as those of Machado et al. [20], in PLA and TPU impregnations with OLE, attributing additionally part of the influence to the decrease in the scCO_2_ density with a higher temperature.

To analyze whether the impregnated compounds confer antioxidant properties to the filaments, antioxidant activity studies were carried out. Figure 6b shows the values of the percentage inhibition of DPPH oxidation for the different conditions of pressure and temperature that were studied. The *DPPH*% *I* varied between 12.3% and 36.1%, for most of the conditions studied, although the results obtained at 250 bar and 75 °C stand out with much higher values of 71.3%.

The competition between the different compounds present in the extract, whether they are antioxidants or not, varies according to the operating conditions during the impregnation process. This competition can manifest in terms of both solubility in the supercritical phase and the ability to access active sites within the polymer matrix. Thus, at the lowest pressure (100 bar), the impregnation of antioxidant substances was affected, providing the lowest values at the three temperature conditions studied. No correlation was observed between the loading of impregnated compounds and the antioxidant activity of the filament. Similar results have been confirmed by Machado et al. [20] for OLE on TPU and PLA or Rosales et al. [59] for mango leaf extract on PLA.

The identification of the impregnated polyphenols evidenced the presence of oleuropein in all selected samples, with values of 54.6, 28.8, and 37 μg/100 mg of polymer when impregnated at 75 °C and 100, 250, and 400 bar, respectively. However, luteolin-7-glucoside was only detected in the sample impregnated at 250 bar and 75 °C with a concentration of 4 μg/100 mg of polymer. These results are in agreement with the higher antioxidant activity reported at 250 bar and 75 °C. It is well known that the antioxidant activity of natural extracts could be the result of the synergistic effect among polyphenols [60].

As with the loading results, an analysis of variance was performed to determine statistically significant influences, as reflected in Table 8.

Analyzing the Pareto diagrams for loading and DPPH antioxidant activity (Table 7 and Table 8), it is observed that both are significantly influenced by the temperature; however, the behavior of pressure is not the same. Therefore, filaments impregnated at 75 °C and the three pressure conditions (100, 250, and 400 bar) were selected to evaluate their performance in SEM and release tests.

**Figure 7:**
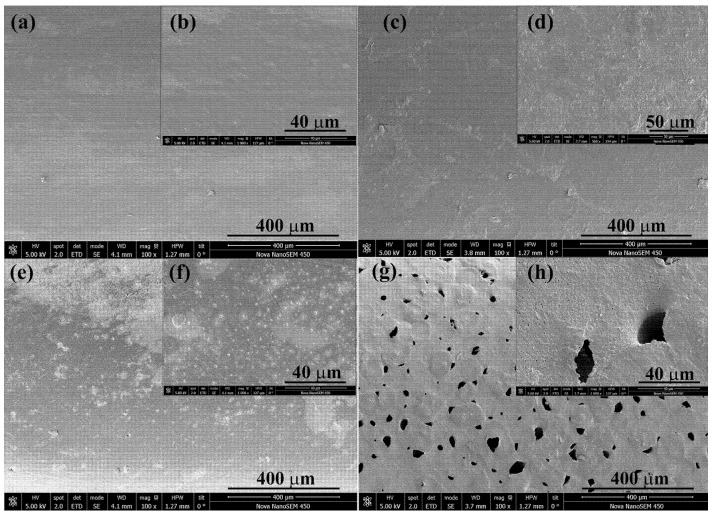
Longitudinal section at 100× and 1000× magnification of control filament (**a**,**b**) and filament impregnated at 100 bar/75 °C (**c**,**d**); 250 bar/75 °C (**e**,**f**) and 400 bar/75 °C (**g**,**h**).

**Figure 8:**
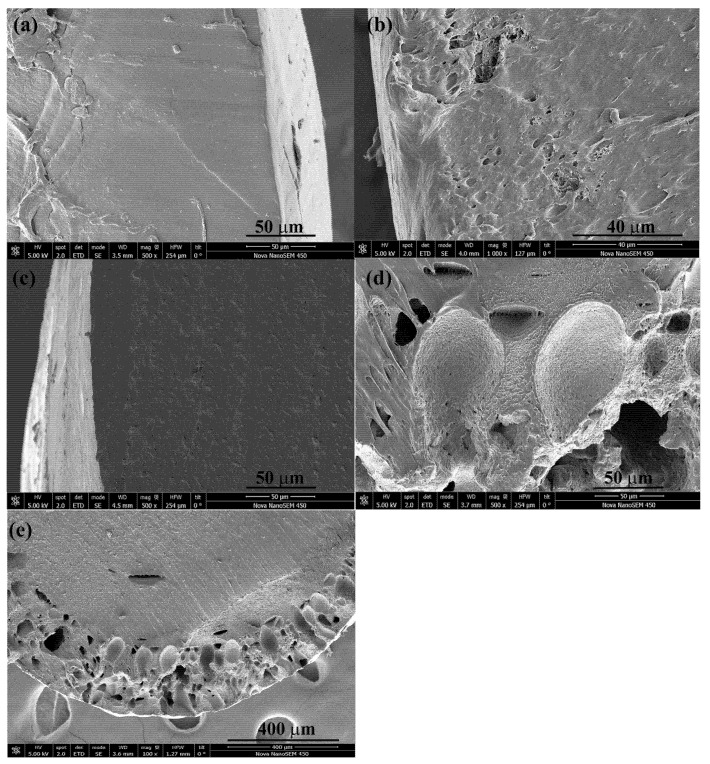
Cross section at 500× magnification of control filament (**a**) and filament impregnated at 100 bar/75 °C (**b**); 250 bar/75 °C (**c**) and 400 bar/75 °C ((**d**,**e**) (100×)).

The scanning electron microscopy analysis revealed that samples impregnated at 250 bar at 75 °C possessed a higher number of superficial particles from the extract than that impregnated at 100 bar (Figure 7c–f), which corroborate the higher antioxidant activity. The compounds impregnated superficially promote the rapid reaction with the DPPH reagent. This fact together with the more complex composition of the samples impregnated at 250 explains the high antioxidant value obtained in that sample. The cross-section of these samples (Figure 8b,c) showed a smooth surface with no porosity that limits the release of the compounds impregnated in the inner parts. On the other hand, samples impregnated at 400 bar showed a different appearance, with irregular pores distributed along the surface of the polymer (Figure 7g,h) and at an external layer of ca. 250 μm (Figure 8d,e), which confirm the swelling results (Figure 5a).

### 3.3. In Vitro Release Studies

**Figure 9:**
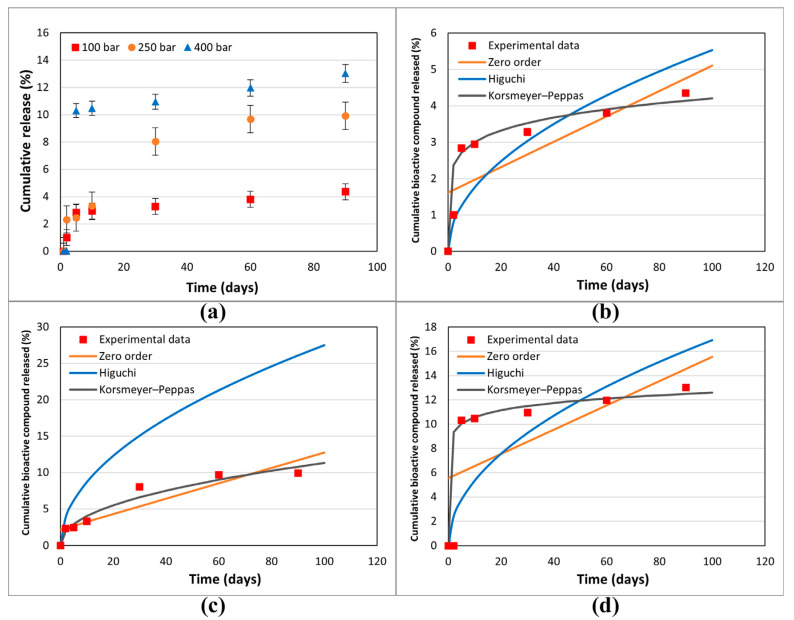
(**a**) Cumulative release (%) of impregnated PCLC filaments at different operation conditions (the results are presented as means and standard deviations (*n* = 3)). Cumulative % bioactive compound release from filaments impregnated at (**b**) 100 bar, (**c**) 250 bar, and (**d**) 400 bar.

To assess the therapeutic efficacy of impregnated polymers, the release kinetics of the active compounds must be analyzed. The main challenge in designing effective controlled-release formulations is maintaining the concentration of the active ingredient within the therapeutic window—i.e., between the minimum effective and minimum toxic concentrations—over a prolonged period [61]. To study the influence of the operating conditions of the impregnation process on the release rate, samples were obtained at 75 °C under three evaluated pressures: 100, 250, and 400 bar, which were selected for analysis. These samples exhibited high loading values and % I, as illustrated in Figure 6a,b. The corresponding release profiles are presented in Figure 9a.

All release curves mainly consisted of two phases, as the first phase, a “burst release”, and then “slow-release”, so that the second phase took place. The initial “burst” release is beneficial for the release of active compounds (anti-inflammatories and antioxidants) as it is essential to inhibit the body’s inflammatory response or eliminate radicals before they can proliferate and/or multiply. The second “slow release” phase provides the prolonged release of the active compounds during the 90-day trial. However, these profiles should be adjusted according to the medical application in which they are to be used.

The filament functionalized at 400 bar with the lowest loading value (1.51%, Figure 6a) exhibits the highest release profile, reaching 13% after 90 days. The high density of scCO_2_ at 400 bar/75 °C (839.93 kg/m^3^) compared to 100 bar/75 °C (233.43 kg/m^3^) increases the solubility of the extract compounds in the supercritical phase and decreases their affinity towards the polymer phase. Consequently, lower loading values are obtained at 400 bar/75 °C than at 100 bar/75 °C. Furthermore, Figure 5a shows that the highest swelling occurs at 400 bar/75 °C, and Figure 7g,h, as well as Figure 8d,e, corroborate the high porosity of these samples. Diffusion-based release can occur either through the polymer matrix itself, where molecules make their way between the polymer chains or through a network of interconnected pores. Therefore, a polymer matrix with higher porosity, such as that obtained at 400 bar/75 °C, offers less resistance to the movement of molecules, resulting in a higher release rate. Conversely, the sample obtained at 100 bar/75 °C is less porous and offers more resistance, affecting the movement of molecules into the PBS medium and resulting in fewer compounds being released.

On the other hand, larger molecules diffuse more slowly than smaller molecules due to the greater difficulty they encounter when moving through the polymeric network. Drugs with a lower molecular weight generally exhibit faster release in aqueous media, such as PBS [62]. An analysis of the released samples via HPLC showed that, at 100 and 400 bar, the main compound released was oleuropein. Oleuropein is an intermediate-sized molecule with a molecular weight of approximately 540.51 g/mol, which could explain these low release values; its solubility in PBS is also limited. Dexamethasone (392.47 g/mol), a widely used drug for treating inflammatory diseases, was used to develop a PLCL-based polymeric device via the hot extrusion method to achieve controlled release of the active ingredient at the implant site. The drug release profile was highly dependent on the dexamethasone/PLCL loading, reaching values below 15% within 28 days [30].

The device functionalized at 250 bar/75 °C exhibits different behaviors to those impregnated at 100 bar/75 °C or 400 bar/75 °C. The higher antioxidant activity value (Figure 6b) may be due to the selective impregnation of active compounds, the synergistic effect of which leads to these high results. Despite the low porosity shown in Figure 7e,f and Figure 8c, the release profiles are intermediate between those achieved at 100 and 400 bar (Figure 9a). An HPLC analysis indicates that oleuropein was not detected in the released samples; instead, the released compounds correspond to quercetin derivatives, which absorb at 340 nm [50].

The release profiles shown in Figure 9a are much higher than those reported for the release of PLA-impregnated mango leaf extract [59]. However, results reported by Cejudo Bastante et al. (2022) [63] for the release of a mango leaf extract impregnated in TPU and PETG, achieve values close to 100% before 10 days. This may be indicative of a pro-funding impregnation of the OLE in the PLCL, which would also explain why the color of the released fragments does not change with respect to the original impregnated polymer.

No visible damage to the polymer structure was observed during the 90-day release study. A recent study on the degradation rate of PLCL in cardiovascular applications showed that this rate can be adjusted over a period of several months, depending on the monomer ratio and the polymer’s molecular weight [64]. Therefore, future research should include rigorous evaluations of the chemical and physical stability of the newly generated material.

The various mathematical models used to describe drug release kinetics provide valuable tools for predicting the release behavior of active compounds from polymeric systems such as PLCL. Zero-order release is characterized by a constant release rate, independent of the concentration of the active compound [62]. The Higuchi model describes a release proportional to the square root of time, typically diffusion-controlled. The Korsmeyer–Peppas model is an empirical model used to determine the release mechanism of polymer matrices, with diffusion, swelling, or erosion, based on the value of the release exponent [65]. The choice of model depends on the specific characteristics of the active compound delivery system and the observed release profile. Table 9 shows the obtained parameters and R^2^ when applying the three studied models. The R^2^ value indicates that the three-impregnation conditions evaluated for release fit the Korsmeyer–Peppas model to a greater extent.

Figure 9b–d show the results achieved by applying the three models mentioned above to each of the operating conditions studied in the release (100, 250, and 40 bar at 75 °C). The graphical representations of the cumulative % release of active compound versus time showed that the polymers followed the Korsmeyer–Peppas model correctly. The *n* < 0.45 value indicates Fickian diffusion, while values between 0.45 and 1.00 suggest non-Fickian. The “*n*” values indicated that the releases of the active compounds followed Fick’s law of diffusion. As a general conclusion, the release of the OLE compounds from PLCL polymers was likely to be controlled by a diffusion mechanism.

**Figure 10:**
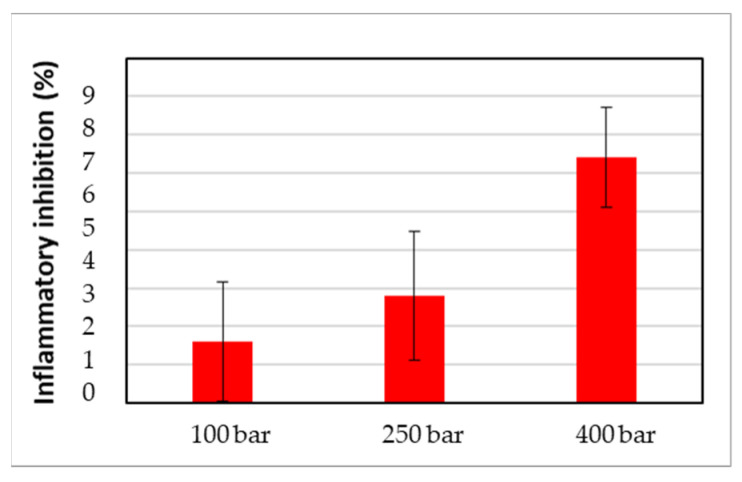
Anti-inflammatory capacity of the 60-day liberation chosen samples. The results are presented as means and standard deviations (*n* = 3).

As shown in Figure 10, the substances released have anti-inflammatory potential; the samples impregnated at 400 bar showed higher values. This finding correlates with the higher release rates observed previously (Figure 9a). However, it is important to note that these results are preliminary, and further research is required.

## 4. Conclusions

This study demonstrates that supercritical impregnation is a viable strategy to functionalize PLCL filaments, thus valorizing olive leaves. Enhanced solvent extraction (CO_2_/ethanol 1:1 *v*/*v*) is effective to obtain olive leaf extracts with high yields and high concentrations of bioactive compounds. The OLEs obtained show high antioxidant and anti-inflammatory activity, with similar behavior for irrigated and dry leaves, confirming their potential for incorporation into materials with biomedical applications.

The supercritical impregnation process in PLCL filaments was significantly affected by the operating parameters; a low depressurization rate was crucial to avoid material deformation. The impregnation pressure and temperature influenced the loading of OLE on the filaments in their resulting antioxidant activity.

The release kinetics of active compounds from PLCL depends on the complex interplay of polymer properties, active compound characteristics, and environmental factors. Studies have shown that active compounds are released over a period of 90 days, which supports the use of the generated devices for applications requiring prolonged exposure to these compounds. The Korsmeyer–Peppas mathematical model is a valuable tool for understanding and predicting these release behaviors.

Despite the progress achieved, there are still many challenges in this field, and future research should focus on studying the mechanical properties of new PLCL-based materials. In addition, in vitro and in vivo studies are essential for evaluating the activity of such impregnated polymers.

## Figures and Tables

**Table 1:** Designs of experiments for the supercritical impregnation of PCLC filaments.

Factor	Variable	Levels	Response Variable	Fixed Parameters
A	Pressure (bar)	100, 250, 400	Swelling (% S)	Impregnation time = 1 h
B	Temperature (°C)	35, 55, 75		High depressurization rate = 2 bar/seg
A	Pressure (bar)	100, 250, 400	Swelling (% S) OLE loading (% L) DPPH antioxidant activity (% I)	Impregnation time = 1 h Low depressurization rate = 0.278 bar/min
B	Temperature (°C)	35, 55, 75		
		

**Table 2:** Characterization of the obtained OLE. The results are presented as means and standard deviations (*n* = 3).

	Dry Land		Irrigated Land	
	scCO_2_	ESE (CO_2_/ethanol 1:1 *v*/*v*)	scCO_2_	ESE (CO_2_/ethanol 1:1 *v*/*v*)
Extraction yield (%)	0.93 ± 0.12	25.5 ± 1.2	0.89 ± 0.11	22.8 ± 1.6
Extract concentration (g/L)	71.7 ± 12.4	68.4 ± 0.2	77.7 ± 6.6	77.0 ± 2.3
Luteolin-7-glucoside concentration (mg/100 g OLE)	n.d. *	45 ± 4	n.d. *	38 ± 3
Oleuropein concentration (mg/100 g OLE)	n.d. *	1480 ± 20	n.d. *	1750 ± 50
Hydroxytyrosol concentration (mg/100 g OLE)	n.d. *	7 ± 2	n.d. *	19 ± 2
DPPH IC_50_ (µg/mL)/(AAI)	578/0.052	33.5/1.07	447/0.074	32.8/1.10
Anti-inflammatory capacity (IC_50_ for 12.7 µM of linoleic acid, µg/mL)	n.d. *	819	n.d. *	734

* n.d. = non-detected.

**Table 3:** ANOVA table for the variables, in the adjusted order, from the linear regression models fitted to DPPH antioxidant activity for OLE from dry and irrigated lands.

	Sum Sq	Df	Mean Sq	F-Value	*p*-Value
OLE Concentration	1.35359	1	1.35359	2242.91	0.0000
y-intercept	0.00041853	1	0.00041853	0.69	0.4190
slope	0.0000151678	1	0.0000151678	0.03	0.8763
Model	1.35402	3			

**Table 4:** ANOVA table for the variables, in the adjusted order, from the linear regression models fitted to the inhibition of inflammation for OLE from dry and irrigated lands.

	Sum Sq	Df	Mean Sq	F-Value	*p*-Value
OLE Concentration	0.597813	1	0.597813	667.08	0.0000
y-intercept	0.00346884	1	0.00346884	3.87	0.0847
slope	0.0015048	1	0.0015048	1.68	0.2312
Model	0.602787	3			

**Table 5:** ANOVA table and Pareto chart for quadratic model of PLCL swelling when impregnated with OLE.

	Sum Sq	Df	Mean Sq	F-Value	*p*-Value	
A: Temperature	399.997	1	399.997	79.09	0.0000	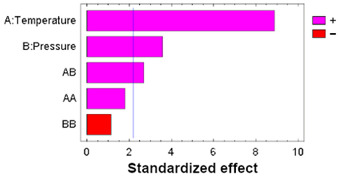
B: Pressure	65.1934	1	65.1934	12.89	0.0042	
AA	16.4295	1	16.4295	3.25	0.0989	
AB	36.6796	1	36.6796	7.25	0.0209	
BB	6.51100	1	6.51100	1.29	0.2806	
Blocks	0.30681	1	0.30681	0.06	0.8100	
Error	55.6294	11	5.05721			
Total	580.726	17				

**Table 6:** ANOVA table and Pareto chart for quadratic model of PLCL swelling when impregnated with OLE at low a depressurization rate.

	Sum Sq	Df	Mean Sq	F-Value	*p*-Value	
A: Temperature	49.8984	1	49.8984	28.96	0.0002	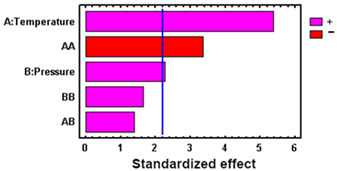
B: Pressure	8.89241	1	8.89241	5.16	0.0442	
AA	19.5217	1	19.5217	11.33	0.0063	
AB	3.31531	1	3.31531	1.92	0.1928	
BB	4.71614	1	4.71614	2.74	0.1262	
Blocks	22.6240	1	22.6240	13.13	0.0040	
Error	18.9503	11	1.72275			
Total	127.918	17				

**Table 7:** ANOVA table and Pareto chart for quadratic model of OLE loading in PLCL.

	Sum Sq	Df	Mean Sq	F-Value	*p*-Value	
A: Temperature	0.53763	1	0.53763	14.40	0.0030	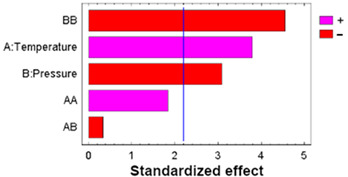
B: Pressure	0.35708	1	0.35708	9.57	0.0102	
AA	0.12721	1	0.12721	3.41	0.0919	
AB	0.00405	1	0.00405	0.11	0.7480	
BB	0.77147	1	0.77147	20.67	0.0008	
Blocks	0.00080	1	0.00080	0.02	0.8863	
Error	0.41061	11	0.03733			
Total	2.20884	17				

**Table 8:** ANOVA table and Pareto chart for quadratic model of antioxidant activity of the impregnated filaments.

	Sum Sq	Df	Mean Sq	F-Value	*p*-Value	
A: Temperature	877.230	1	877.230	5.60	0.0374	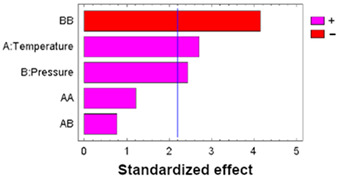
B: Pressure	711.480	1	711.480	4.54	0.0565	
AA	397.338	1	397.338	2.54	0.1396	
AB	71.4013	1	71.4013	0.46	0.5136	
BB	1510.62	1	1510.62	9.64	0.0100	
Blocks	115.520	1	115.520	0.74	0.4089	
Error	1723.87	11	156.716			
Total	5407.46	17				

**Table 9:** Model parameters and R-squared values.

	Zero Order		Higuchi		Korsmeyer–Peppas		
	K_0_	R^2^	K_H_	R^2^	k_KP_	n	R^2^
100 bar/75 °C	0.0349	0.6004	0.5528	0.7079	2.1419	0.1464	0.9108
250 bar/75 °C	0.1058	0.8054	2.748	0.8952	1.4231	0.4403	0.9362
400 bar/75 °C	0.1007	0.4521	1.6929	0.6163	8.8746	0.0759	0.8901

## Data Availability

The original contributions presented in this study are included in the article. Further inquiries can be directed to the corresponding author.
